# Identification of stable quantitative trait loci (QTLs) for fiber quality traits across multiple environments in *Gossypium hirsutum* recombinant inbred line population

**DOI:** 10.1186/s12864-016-2560-2

**Published:** 2016-03-08

**Authors:** Muhammad Jamshed, Fei Jia, Juwu Gong, Koffi Kibalou Palanga, Yuzhen Shi, Junwen Li, Haihong Shang, Aiying Liu, Tingting Chen, Zhen Zhang, Juan Cai, Qun Ge, Zhi Liu, Quanwei Lu, Xiaoying Deng, Yunna Tan, Harun or Rashid, Zareen Sarfraz, Murtaza Hassan, Wankui Gong, Youlu Yuan

**Affiliations:** State Key Laboratory of Cotton Biology, Key Laboratory of Biological and Genetic Breeding of Cotton, The Ministry of Agriculture, Institute of Cotton Research, Chinese Academy of Agricultural Sciences, Anyang, 455000 Henan China; College of Agronomy, Xinjiang Agricultural University, Key Laboratory of Agro-Biotechnology, Urumqi, 830052 Xinjiang China; College of Bioscience and Biotechnology, Hunan Agricultural University, Changsha, 410128 Hunan China; Anyang College of Technology, Anyang, 455000 Henan China; Department of Materials Science and Engineering, College of Engineering, Peking University, Beijing, 100871 China

**Keywords:** Recombinant inbred line, Upland cotton, Multiple environments, SSR markers, Meta-QTL analyses, Stable QTLs

## Abstract

**Background:**

The identification of quantitative trait loci (QTLs) that are stable and consistent across multiple environments and populations plays an essential role in marker-assisted selection (MAS). In the present study, we used 28,861 simple sequence repeat (SSR) markers, which included 12,560 *Gossypium raimondii* (D genome) sequence-based SSR markers to identify polymorphism between two upland cotton strains 0–153 and sGK9708. A total of 851 polymorphic primers were finally selected and used to genotype 196 recombinant inbred lines (RIL) derived from a cross between 0 and 153 and sGK9708 and used to construct a linkage map. The RIL population was evaluated for fiber quality traits in six locations in China for five years. Stable QTLs identified in this intraspecific cross could be used in future cotton breeding program and with fewer obstacles.

**Results:**

The map covered a distance of 4,110 cM, which represents about 93.2 % of the upland cotton genome, and with an average distance of 5.2 cM between adjacent markers. We identified 165 QTLs for fiber quality traits, of which 47 QTLs were determined to be stable across multiple environments. Most of these QTLs aggregated into clusters with two or more traits. A total of 30 QTL clusters were identified which consisted of 103 QTLs. Sixteen clusters in the A_t_ sub-genome comprised 44 QTLs, whereas 14 clusters in the D_t_ sub-genome that included 59 QTLs for fiber quality were identified. Four chromosomes, including chromosome 4 (c4), c7, c14, and c25 were rich in clusters harboring 5, 4, 5, and 6 clusters respectively. A meta-analysis was performed using Biomercator V4.2 to integrate QTLs from 11 environmental datasets on the RIL populations of the above mentioned parents and previous QTL reports. Among the 165 identified QTLs, 90 were identified as common QTLs, whereas the remaining 75 QTLs were determined to be novel QTLs. The broad sense heritability estimates of fiber quality traits were high for fiber length (0.93), fiber strength (0.92), fiber micronaire (0.85), and fiber uniformity (0.80), but low for fiber elongation (0.27). Meta-clusters on c4, c7, c14 and c25 were identified as stable QTL clusters and were considered more valuable in MAS for the improvement of fiber quality of upland cotton.

**Conclusion:**

Multiple environmental evaluations of an intraspecific RIL population were conducted to identify stable QTLs. Meta-QTL analyses identified a common chromosomal region that plays an important role in fiber development. Therefore, QTLs identified in the present study are an ideal candidate for MAS in cotton breeding programs to improve fiber quality.

**Electronic supplementary material:**

The online version of this article (doi:10.1186/s12864-016-2560-2) contains supplementary material, which is available to authorized users.

## Background

Cotton (Genus *Gossypium*) is a well-known and highly important industrial crop that has been grown in more than 80 countries located in tropical and subtropical regions [1]. It is used as an important source of natural fiber, seed oil and proteins [2]. The genus *Gossypium* comprises approximately 45 diploid species and five tetraploid species. Two tetraploid species, *G. hirsutum* and *G. barbadense,* and two diploid species, *G. herbaceum* and *G. arboreum* have been extensively cultivated around the world, with *G. hirsutum* covering >90 % of the total world production and is generally referred to as upland cotton [3]. Upland cotton has a high yield potential, whereas *G. barbadense* has superior fiber quality attributes that subsequently gives it a 30–50 % price advantage over upland cotton [4], whereas the low yield and poor adaptation of *G. barbadense* restricts its production to specific regions around the world. To fulfill the global requirements of the growing human population and the recent advancement in spinning technology justify the need for increased cotton fiber yield and improved cotton fiber traits. Fiber quality traits and yield components are quantitative traits that are negatively correlated [5]. Therefore, it is very difficult to improve all these traits simultaneously by using conventional breeding procedures. Moreover, this would also be laborious and time consuming [6].

Marker-assisted selection (MAS) is prestigious blessing that breaks the linkage among these traits, as it directly selects genetic markers that are tightly linked to quantitative trait loci (QTLs) other than the conventional procedure of indirectly selecting strains with superior phenotypic performance for breeding. Recent developments in field of molecular markers have allowed plant breeders to identify and evaluate complex agronomical traits. The construction of a molecular genetic map is a foundation for the genetic dissection of important economical and agronomical traits, MAS, and map-based cloning [7]. The first molecular linkage map was constructed in 1994 [8]. Since then, several genetic maps have been constructed including interspecific [9–14] and intraspecific crosses [15–20], to explore the cotton genome and to identify QTLs. However, most fiber QTLs obtained from interspecific crosses have limited applications to upland cotton breeding programs [21, 22] as most of markers used in interspecific cross do not show polymorphism in intraspecific crosses [23]. Saturated intraspecific upland cotton maps are useful but more challenging to construct because of the markedly low rate of polymorphisms of molecular markers within *G. hirsutum*. To overcome this obstacle scientists have employed different mapping populations or used whole-genome sequence-based markers. They used populations involving more than two parents, which have higher polymorphism rates in intraspecific crosses, namely, from 6.6 to 13.7 %, thereby ensuring a surge in genetic diversity and facilitating the identification of more QTLs [19, 23, 24].

Recently physical genome drafts of *G. raimondii* [25, 26] *G. arboreum* [27] and *G. hirsutum* [28, 29] have been completed which could be utilized in the construction of a high-density linkage map and investigate complex traits such as fiber quality. A previous study suggested that the tetraploid species originated from the hybridization of two diploid species, *G. arboreum* (A genome) and *G. raimondii* (D genome) about 1–2 million years ago [2]. Furthermore, more QTLs for fiber traits have been mapped to the D_t_ sub-genome of upland cotton compared to that in the A_t_ sub-genome, thus suggesting that it may play an important role in fiber developments [30–32]. A high-coverage genetic map constructed by Tang et al. [33] with SSR markers developed from *G. raimondii* BAC-end sequences has revealed that these D genome-based primers are widely distributed and suitable for whole-genome mapping. Therefore, because of the importance of the D_t_ sub-genome in determining fiber quality traits [23], we used D genome (*G. raimondii*) sequence-based SSR primers [26], together with SSR primers from Cotton Marker Database (http://www.cottonmarker.org/) to construct an intraspecific linkage map. Previously, Sun et al. [18] reported a linkage map based on an intraspecific cross of upland cotton cultivars sGK9708 and 0–153. They used 200 SSR markers to construct a genetic map and identified 50 QTLs for fiber quality in the F_2_, F_2:3_ and RIL populations in 4 environments. We added 603 primers to our published genetic map and identified QTLs for fiber quality in 11 environments, including four previously reported environments [18] (Table 1) to augment our previous results from the same intraspecific RIL (F_6:8_) population of upland cotton. Furthermore we conducted a meta-analyses with Biomercator V4.2 [34] using the fiber QTLs identified from the present study, those previously reported in F_2_, F_2:3_ and RIL population [18], and those generated from meta-analyses conducted by Said et al. [35, 36], along with three succeeding QTLs studies [33, 37, 38]. We identified some stable and consistent QTLs that aggregated into clusters in upland cotton. These QTL clusters can be made more valuable to MAS to improve the fiber quality of upland cotton.

**Table 1:** Details of 11 environments used to evaluate 196 RIL along with their parents

Year	Environment	Abbreviation used	Replication	Layout
2007	Anyang^a^	Ay07	2	5× 0.8 m
2008	Anyang^a^	Ay08	2	5× 0.8 m
	Quzhou^a^	Qz08	2	5× 0.8 m
	Linqing^a^	Lq08	2	5× 0.8 m
2009	Anyang	Ay09	2	5× 0.8 m
	Quzhou	Qz09	2	5× 0.8 m
	Akesu	Ak09	2	2× 0.6 m
2010	Anyang	Ay010	2	5× 0.8 m
	Zhengzhou	Zz010	2	5× 0.8 m
	Gaoyi	Gy010	2	5× 0.6 m
2013	Anyang	Ay013	2	5× 0.8 m

^a^Data of these environments was reported in our previous report and used for QTL mapping but excluded in ANNOVA except Quzhou 2008

## Results

### Assessment of phenotypic performance

The phenotypic performance of the five fiber traits was observed to continuously segregate, and transgressive segregation was observed. Very low absolute skewness and kurtosis values showed that these traits were normally distributed (Table 2). The results of correlation analyses of fiber quality traits in RILs are presented in Table 3. Positive correlations between any of the two traits, which included fiber elongation (FE), fiber length (FL), fiber strength (FS), and fiber uniformity (FU), were observed, with a significance level of 0.01. Fiber micronaire (FM) was negatively correlated with FL and FS. ANNOVA revealed that fiber quality traits presented significant environmental and genetic effects (*P* < 0.01, Table 4). A broad sense heritability test was also performed for all fiber traits as defined elsewhere [39]. Fiber elongation had the lowest heritability (0.27), whereas that of other fiber traits was high, ranging from 0.80 (FU) to 0.93 (FL).

**Table 2:** The observed phenotypic performance of mean values of fiber quality traits of two parents and RILs in 11 environments

Trait^a^	0-153	sGK9708	RIL	Minimum	Maximum	Std. deviation	Kurtosis	Skewness
FL	30.25	27.40	29.20	23.81	33.69	1.288	−0.256	−0.059
FU	85.66	83.25	84.50	77.10	88.05	0.845	0.096	−0.318
FM	4.39	4.90	4.37	2.21	6.57	0.395	0.078	−0.080
FE	6.49	6.38	6.45	5.55	7.50	0.058	0.357	−0.181
FS	33.27	25.75	30.05	22.85	36.10	1.872	0.092	0.293

^a^ *FL* fiber length, *FU* fiber uniformity, *FM* fiber mironaire, *FE* fiber elongation, *FS* fiber strength

**Table 3:** Correlation analyses among fiber quality traits based on eleven environments for RIL

Traits^a^	FL	FU	FM	FE
FU	0.616^b^			
FM	−0.371^b^	0.031		
FE	0.487^b^	0.619^b^	−0.023	
FS	0.740^b^	0.759^b^	−0.364^b^	0.592^b^

^a^For trait abbreviations see Table 2

^b^Indicates the correlation reaches the significant level at 0.01

**Table 4:** ANNOVA and a broad sense heritability of fiber quality traits in RIL population

Traits^a^	Source	DF	Mean square	*F* Value	Pr > F	H^2^_B_
FL	e	7	178.2	266.85	<.0001	0.93
	g	195	23.2	34.81	<.0001	
	g*e	1365	1.4	2.15	<.0001	
FU	e	7	364.9	298.18	<.0001	0.8
	g	195	11.6	9.47	<.0001	
	g*e	1365	2.0	1.63	<.0001	
FM	e	7	32.0	200.99	<.0001	0.85
	g	195	2.5	15.74	<.0001	
	g*e	1365	0.3	2.11	<.0001	
FE	e	7	217.9	4905.24	<.0001	0.27
	g	195	0.2	4.62	<.0001	
	g*e	1365	0.2	3.97	<.0001	
FS	e	7	1582.2	1056.22	<.0001	0.92
	g	195	44.9	30.01	<.0001	
	g*e	1365	3.7	2.49	<.0001	

^a^For trait abbreviations see Table 2

H^2^_B_ is broad sense heritability, e is environment and g is genotype

### Construction of a genetic map

In the present study, we obtained 851 primer pairs that were clearly polymorphic between the two parents, 0–153 and sGK9708. These 851 primer pairs generated 997 loci, in which 132 pairs produced two loci, 13 pairs yielded three loci, and two pairs resulted in four loci. All 997 loci were used in the construction of a linkage map. A total of 793 loci were grouped into 76 linkage groups. Seventy three groups were assigned to 26 chromosomes of upland cotton (Additional file 1). Three groups could not be associated with any chromosome. We named these “UD” following the number. The total recombinant length of this map was 4,110 cM, which represented approximately 93.2 % [40] of the total length of the cotton genome, with an average distance of 5.2 cM between adjacent markers. The A_t_ sub-genome spanned 1,635 cM, consisted of 269 markers on 37 linkage groups, and with an average distance of 6.1 cM between adjacent markers. Thirty six groups were assigned to the D_t_ sub-genome and comprised 524 markers spanning 2,327.4 cM, with an average of 4.6 cM between adjacent loci (Table 5). Chromosomes c4, c5, c14, c16 and c25 had more markers compared to the other chromosomes. Among these, c25 had 113 loci that encompassed204 cM, with an average distance of 1.9 cM between two adjacent markers. The smallest group, c11, had 8 markers, and a total length of 37.8 cM.

**Table 5:** Genomic distributions of SSR markers and identified QTLs

Chromosome	Linkage groups	Mapped markers	Distorted loci	Total distance covered	Aver. distance b/w markers	Max^a^. distance b/w markers	Min^a^. distance b/w markers
Chr1	3	17	6	158.2	9.0	18.4	2.09
Chr2	3	23	12	106.6	3.9	18.0	0.37
Chr3	2	15	5	108.4	11.3	28.7	0.68
Chr4	1	36	15	177.4	5.1	25.8	0.51
Chr5	2	31	9	170.6	6.3	27.9	0.50
Chr6	4	35	7	193.6	3.6	14.8	0.42
Chr7	3	25	14	103.8	4.3	12.7	0.40
Chr8	2	11	2	99.8	11.0	16.8	4.76
Chr9	5	14	3	132.6	13.0	15.9	11.44
Chr10	4	14	6	96.5	21.5	30.4	12.56
Chr11	3	8	3	37.9	9.5	17.5	1.60
Chr12	4	21	9	170.8	10.3	25.0	0.45
Chr13	1	19	9	79.2	4.4	16.4	0.35
Chr14	3	55	22	200.1	3.1	21.5	0.19
Chr15	2	41	13	161.2	3.3	14.9	0.36
Chr16	1	67	34	132.3	2.0	9.8	0.12
Chr17	4	18	9	149.9	7.6	20.2	1.73
Chr18	5	39	23	268.5	4.6	24.8	1.07
Chr19	3	26	11	170.1	4.2	14.5	0.17
Chr20	4	33	21	217.3	7.6	32.0	0.34
Chr21	2	28	14	213.3	6.0	16.6	1.73
Chr22	2	21	6	126.1	7.0	13.5	2.38
Chr23	3	39	12	224.4	5.7	20.7	0.78
Chr24	1	14	12	108.7	8.4	31.8	0.38
Chr25	1	113	62	204.8	1.9	13.8	0.01
Chr26	5	18	12	150.8	7.9	14.7	2.88
UD	3	12	10	147.3	10.8	21.4	0.08
Total (AD)	76	793	361	4110.0	5.2	32.0	0.01

^a^ Max. Distance means maximum marker interval within linkage groups in that chromosome and Min distance means minimum marker interval between two markers in linkage groups of particular chromosome

### Segregation distortion of SSR markers

Segregation distortion is a common occurrence in plants [41], including cotton [7]. We observed severe segregation distortions at a rate of about 45 % (Table 5). Among the 361 distorted loci, 241 (67.1 %) favored sGK9708 alleles and 119 (32.9 %) involved 0–153 alleles. A total of 36 segregation distortion regions (SDRs) were detected on 20 chromosomes (Additional file 1). The A_t_ sub-genome contained 10 SDRs, whereas the D_t_ sub-genome comprised 26 SDRs. The largest SDR was on c25, which consisted of 26 distorted loci. The highest number of SDRs on one chromosome was 5, which was observed in c16 and c25. One chromosome (c21) contained 3 SDRs, 6 chromosomes (c4, c13, c14, c18, c20, and c26) comprised 2 SDRs, whereas the remaining 11 chromosomes (c2, c5–c9, c15, c17, c19, c23, and c24) harbored only 1 SDR.

### Collinearity between the linkage and physical map

**Fig. 1:**
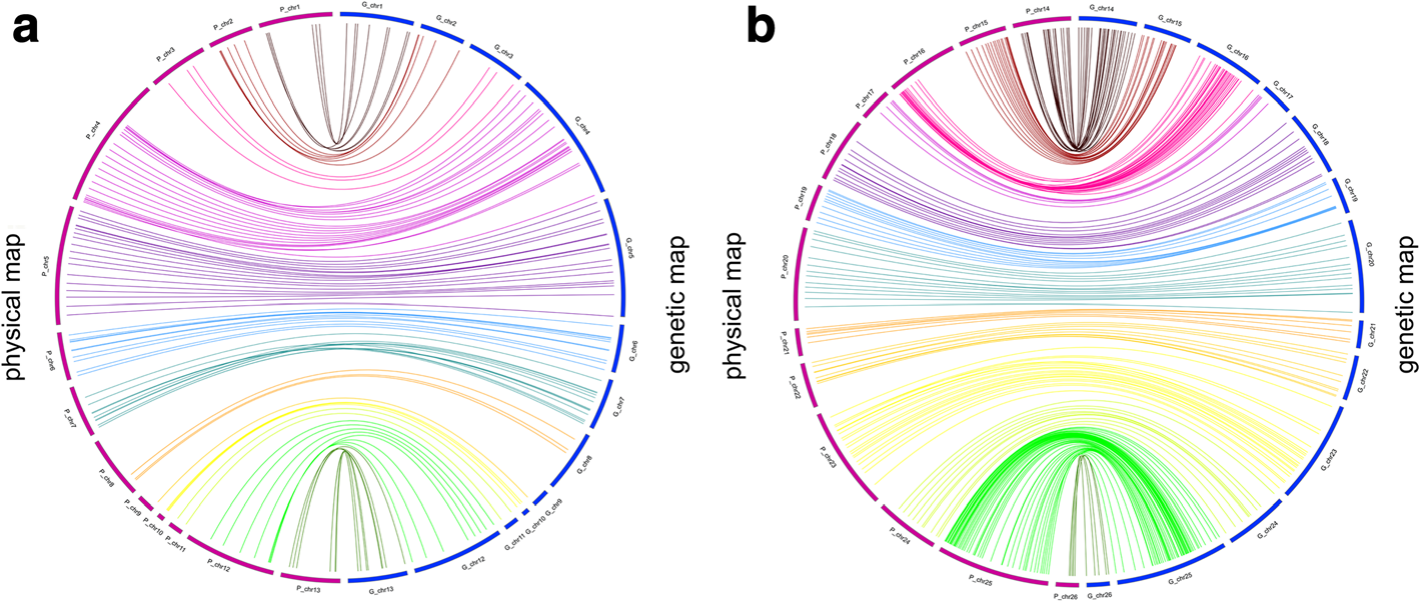
Collinearity analyses between genetic map 0–153 and physical map of *G. hirsutum*. **a** Collinearity analyses between genetic map of 0–153 from C1-C13 (total distance 1635 cM) with corresponding sequence on At sub-genome (1.16GB) of *G. hirsutum*. **b** Collinearity analyses between C14-C26 (total distance 2327 cM) of genetic map with corresponding sequence of D_t_ sub-genome (776 Mb) of *G. hirsutum*

Loci collinearity between linkage map and the *G. hirsutum* physical map of various chromosomes is presented in Fig. 1. Some loci whose physical location was not confirmed were excluded from the analysis. The overall loci order on the genetic map was in agreement with the order of corresponding sequences on the A_t_ and D_t_ sub-genomes of *G. hirsutum*. In the A_t_ sub-genome (c1–c13), 1.76 GB corresponded to 1,635 cM, whereas in the D_t_ sub-genome (c14–c26) 774 Mb was equivalent to 2,327 cM.

### QTL mapping of fiber quality traits

A total of 165 QTLs for five fiber traits were identified on 24 chromosomes using the composite interval mapping method [42]., Forty seven QTLs identified in a minimum of 3 and a maximum of 10 environments were declared as stable QTLs, of which 12 QTLs were described as stable in our previous report [18], whereas 35 were novel. The physical map was also used to identify QTLs that confirmed 69 QTLs, including 43 stable ones. Two chromosomes, c14 and c25 had more QTLs. No QTL was detected on c1 and c8. Approximately 58 QTLs were identified on the A_t_ sub-genome chromosomes, whereas 107 QTLs were localized to the D_t_ sub-genome chromosomes. QTLs positions with their observed phenotypic variance (PV) and nearest loci are listed in Additional file 2 and graphically presented in Additional file 1.

### Fiber length

In total, 31 QTLs for FL were detected on 11 chromosomes, including c4, c6, c7, c14, c16, c18, c21, c22, c23, c24, and c25 (Additional file 3). The highest number of QTLs on one chromosome was 6 (c25). Four chromosomes, c6, c16, c22, and c24, harbored only one QTL. Twelve QTLs for FL were identified in only one environment and 5 QTLs were detected in two environments. Fourteen QTLs were identified in 3 or more environments and declared as stable QTLs. Nine stable QTLs for FL on c4, c7, c16, c23 and c25 have favorable alleles from parent 0–153, whereas 5 stable QTLs on c14, c18 and c21 showed favorable alleles from parent sGk9708. The QTL on c4, *qFL-C4-2,* was identified in three environments, explaining 5.8–8.1 % of the observed PV. Two QTLs on c7, *qFL-C7-1* and *qFL-C7-2* were also identified in 4 environments described in our previous report [18]. The QTL *qFL-C7-1* was stable and identified in 3 environments, explaining 5.8–12.1 % of the observed PV. Three QTLs on c14, *qFL-C14-1, qFL-C14-2* and *qFL-C14-3* were identified in 8, 6, and 3 environments, explaining 8.1–13.1 %, 7.1–11.5 %, and 6.3–8.1 % of the observed PVs, respectively. The QTL *qFL-C14-2* was also identified in our previous report [18] in 3 environments. The QTL on c16, *qFL-C16-1* was identified in three environments, explaining 5.7–7.5 % of the observed PV. The QTL on c18, *qFL-C18-3* was identified in a single environment in our previous report [18] and now in four environments, explaining 5.2–11.0 % of the detected PV. The QTL on c21, *qFL-21-1* was identified in seven environments, explaining 8.7–23.6 % of the observed PV. The QTL on c23, *qFL-C23-2* was identified in a single environment in our previous report [18] and now in three environments, explaining 9.0–14.9 % of the observed PV. Five QTLs on c25, *qFL-C25-2, qFL-C25-3, qFL-C25-4, qFL-C25-5,* and *qFL-C25-6* were respectively identified in 4, 6, 5, 5, and 3 environments, explaining 5.2–10 %, 6.8–9.4 %, 6.8–11.8 %, 6.5–10.5 % and 8.6–10.6 % of the observed PVs, respectively. Two QTLs, *qFL-C25-2* and *qFL-C25-3,* were also previously identified in four environments [18]. In total, 14 QTLs out of 31 were also identified during QTL analysis with the physical map including 11 stable QTLs.

### Fiber strength

A total of 35 QTLs for FS were identified on 13 chromosomes including c4, c6, c7, c9, c11, c12, c13, c14, c18, c19, c21, c23, and c25 (Additional file 3). The highest number of QTLs on one chromosome was 7 (c25). Five chromosomes, c6, c9, c11, c12, and c19, harbor a single QTL. Twenty-one QTLs for FS were identified in only one environment and six QTLs were identified in two environments. Eight QTLs were detected in three or more environments and declared as stable QTLs. Six stable QTLs for FS on c7 and c25 have favorable alleles from parent 0–153, whereas two stable QTLs on c14 showed favorable alleles from parent sGk9708. The QTL on c7, *qFS-C7-1,* was identified in 10 environments, explaining 12.2–26.7 % of the observed PV. The QTL *qFS-C7-2* was identified in seven environments, explaining 7.9–11.2 % of the observed PV. Both stable QTLs were also previously identified in four environments [18]. The QTL on c14, *qFS-C14-3* was identified in eight environments, explaining 4.9–13.7 % of the observed PV. The QTL *qFS-C14-4* was identified in four environments explaining 5.4–8.5 % of the detected PV. Four QTLs on c25, *qFS-C25-3, qFS-C25-4, qFS-C25-5,* and *qFS-C25-6,* were respectively identified in 3, 5, 6 and 7 environments, explaining 7.9–17.0 %, 8.4–15.0 %, 5.4–15.0 %, and 6.4–15.8 % of the observed PVs. Two QTLs, *qFS-C25-3* and *qFS-C25-4* were also earlier identified in four environments [18]. All eight stable QTLs were also detected and confirmed through physical map analysis.

### Fiber elongation

For the FE trait, 32 QTLs were identified and located on 13 chromosomes including c3, c4, c7, c10, c13, c14, c15, c19, c21, c22, c23, c25, and c26, explaining 3.15–17.9 % of the observed PV (Additional file 3). The highest number of QTLs on one chromosome was 8 (c25). Six chromosomes, c10, c13, c21, c22, c23, and c26, harbored a single QTL. Eighteen QTLs were identified in one environment, whereas four QTLs were identified in two environments. Ten QTLs for FE were detected and described as stable QTLs. Six stable QTLs for FE on c4, c22, and c25 have favorable alleles from parent 0–153, whereas four stable QTLs on c14 showed favorable alleles from parent sGk9708. Two QTLs on c4, *qFE-C4-2* and *qFE-C4-3* were respectively identified in three and five environments, explaining 4.6–8.5 % and 5.5–12.4 % of the observed PVs, respectively. The QTL, *qFE-C4-2* was also previously identified in four environments [18]. Four QTLs on c14 *qFE-C14-1, qFE-C14-2, qFE-C14-3,* and *qFE-C14-4* were respectively identified in 4, 3, 4 and 3 environments, explaining 8.8–17.9 %, 7.4–14.3 %, 8–15 % and 9.8–11.8 % of the observed PVs. The QTL on c22, *qFE-C22-1,* was identified in three environments, explaining 7.2–13.8 % of the observed PV. Three stable QTLs on c25, *qFE-C25-4, qFE-C25-5,* and *qFE-C25-6* were identified in 3, 4, and 3 environments, explaining 5.6–9.4 %, 5.6–10.1 % and 6.8–10.4 % of the observed PVs, respectively. The QTL *qFE-C25-4* was also earlier identified in three environments [18]. In total, 13 QTLs, including 10 stable ones were also identified and confirmed through physical map-based QTL analysis.

### Fiber uniformity

For FU, 32 QTLs were identified and located on 14 chromosomes including c2, c4, c5, c6, c7, c10, c12, c13, c14, c16, c18, c19, c23, and c25, explaining 1.8–18.2 % of the observed PV (Additional file 3). The highest number of QTLs on one Chromosome was 7 (c25). Seven chromosomes, c5, c6, c12, c13, c18, c19, and c23 harbored a single QTL. Twenty QTLs were identified in one environment, whereas seven 7 QTLs were detected in two environments. Five QTLs for FU were identified as stable QTLs. Three stable QTLs for FU on c7, c13, and c25 have favorable alleles from parent 0–153, whereas two stable QTLs on c14 showed favorable alleles from parent sGk9708. The QTL on c7, *qFU-C7-1* was identified in six environments, explaining 7.0–18.2 % of the observed PV. This was also previously identified in the F_2:3_ and RIL populations in two environments [18]. The QTL on c13, *qFU-C13-1* was identified in three environments, explaining 4.4–6.5 % of the observed PV. It was same QTL that we earlier identified in two environments [18]. Two stable QTLs on c14, *qFU-C14-2* and *qFU-C14-3,* were respectively identified in five and four environments, explaining 6.7–14.2 % and 7.6–10.1 % of the observed PVs. The QTL on c25, qFU*-C25-5* was identified in four environments, explaining 6.4–8.0 % of the observed PV. This was also earlier identified in four environments [18]. In total, 13 QTLs including five stable ones were also confirmed through QTL analysis using a physical map.

### Fiber micronaire

A total of 35 QTLs were identified for FM on 16 chromosomes including c3, c4, c5, c6, c7, c10, c13, c14, c15, c16, c17, c20, c21,c23, c24, and c25 (Additional file 3). The highest number of QTLs on one chromosome was 6 (c25). Eight chromosomes, c3, c7, c10, c13, c17, c21, c23, and c24 harbored a single QTL. Eighteen QTLs were identified in one environment, whereas seven QTLs were identified in two environments. Ten QTLs were identified as stable QTLs. Three stable QTLs on c3, c4 and c16 have favorable alleles from parent 0–153, whereas seven stable QTLs on c7, c14 and c25 comprised favorable alleles from parent sGk9708. The QTL on c3, *qFM-C3-1* was identified in three environments, explaining 5.3–5.6 % of the observed PV. It was also identified in our previous report in one environment [18]. The QTL on c4, *qFM-C4-2* was identified in four environments, explaining 7.7–8.7 % of the observed PV. The QTL on c7, *qFM-C7-1,* was identified in five environments, explaining 9.6–16.7 % of the observed PV. The QTL on c16, *qFM-C16-3,* was identified in three environments, explaining 5.2–7.9 % of the observed PV. It was also identified in our previous report [18] in one environment. Two QTLs on c14, *qFM-C14-2* and *qFM-C14-3,* were identified in four environments, explaining 6.1–9.1 % and 6.5–8.6 % of the observed PVs, respectively. The QTL, *qFM-C14-3* was also identified in our previous report [18] in one environment. Four QTLs on c25, *qFM-C25-1, qFM-C25-2*, *qFM-C25-4,* and *qFM-C25-5,* were respectively identified in 4, 5, 3, and 4 environments explaining 7.3–10.3 %, 5.2–9.9 %, 6.3–8.5 % and 6.2–10.5 % of the observed PVs. The QTL, *qFM-C25-4* was also previously identified [18] in four environments. In total, 15 QTLs, including eight stable ones, were also identified and confirmed through physical map analysis.

### QTL clusters and meta-analysis

QTL clustering is a common phenomenon in plants and also observed in cotton [32, 43, 44]. We identified 30 clusters on 11 chromosomes including c3, c4, c6, c7, c10, c12, c13, c14, c16, c21 and c25. Most of stable QTLs fall in these cluster regions. Six clusters having QTLs for all five fiber traits were identified on c7, c14 and c25, among which, the cluster on c7 *c7-cluster 1* contained five QTLs that were tightly linked to markers PGML00802 and NAU2627 explaining 5.9–26.7 % of the observed PV. Two QTL clusters on c25, *c25-cluster 2* and *c25-cluster 4* contained nine and five QTLs that were tightly linked to markers TMK19, BNL3806b, PGML00463b, SWU19198, and NBRI1529 explaining 5.5–17.0 % and 6.2–14.5 % of the observed PVs, respectively. Three clusters on c14, *c14-cluster 2, c14-cluster 3,* and *c14-cluster 4* each contained five QTLs that were tightly linked to marker SWU14535, PGML00989, NAU3393, SWU 14507, CSHES150, BNL3099, and COT99 and explaining 5.7–15 %, 5.4–11.8 % and 6.3–10.0 % of the observed PVs, respectively. The details of each cluster are summarized in Additional file 4.

**Fig. 2:**
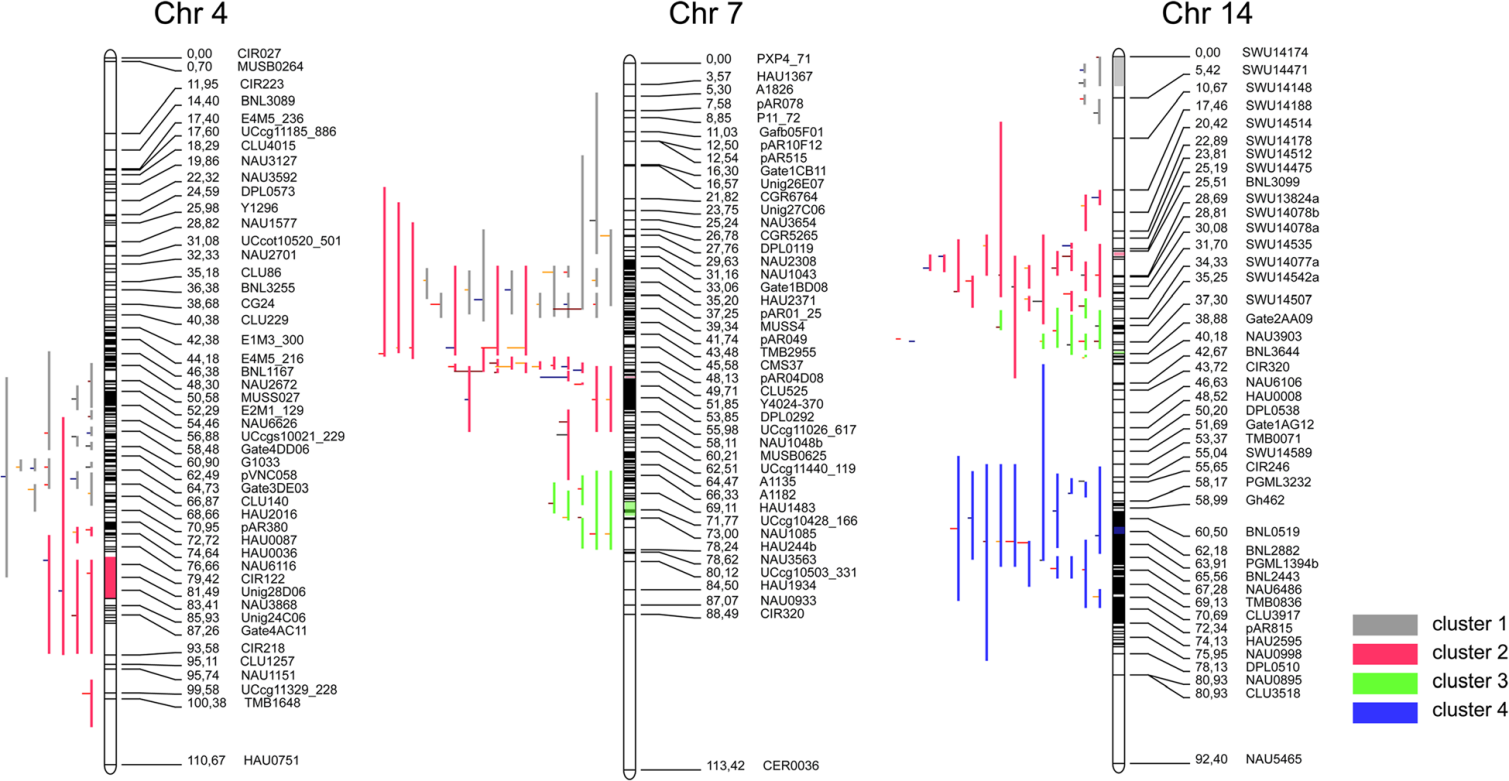
Result of Meta analyses by Biomercator 4.2. QTLs belong to same cluster regions have same color. Length of each QTL vertically represents the confidence intervals. Consensus Chromosome 4 (Cons.c4) has two clusters, Cons.c7 has 3 and Cons.c14 has 4 clusters

In the meta-analysis, a total of 38 meta-cluster regions on 11 chromosomes were identified, which included c4, c5, c7, c12, c13, c14, c15, c16, c20, c23, and c25 (Additional file 5). The results showed that some clusters in the 0-153хsGK9708 genetic map, (which were very close) were grouped into the same 20-cM meta-cluster region on the consensus map and part of same meta-cluster (Additional files 6 and 7). Twenty-nine QTLs were projected on consensus chromosome 4 (Cons.c4), which resulted into 2 QTL meta-clusters*. C4-m-cluster-1* has 14 QTLs, while *C4-m-cluster-2* has seven QTLs (Fig. 2). Fifty-three QTLs were projected on Cons.c7 which yielded three QTL clusters. *C7-m-cluster-1*, *C7-m-cluster 2,* and *C7-m-cluster-3* contained 12, 21 and 6 QTLs respectively (Fig. 2). Seventy-six QTLs were projected on Cons.c14 which resulted in four meta-clusters. C*14-m-cluster-1, C14-m-cluster-2*, *C14-m-cluster-3,* and *C14-m-cluster-4* contained 5, 16, 8, and 15 QTLs respectively (Fig. 2). Sixty-eight QTLs were projected on Cons.c25 which resulted in four QTL clusters. *C25-m-cluster-1*, *C25-m-cluster-2*, *C25-m-cluster-3,* and *C25-m-cluster-4* contained 18, 15, 21, and 6 QTLs, respectively. The details of the remaining QTLs are summarized in Additional file 5. The cluster on Cons.c4, *C4-m-cluster-1* from the 45–65 cM interval was situated between markers DPL0196 and NAU3093 (40,406,319–59,166,290 bp). Cluster, *C4-m-cluster-2* from 73 to 93 cM interval was located between markers DPL0451 and CIR218 (60,349,199–62,668,683 bp). The cluster on Cons.c7, *C7-m-cluster-1,* from the 20–36 cM interval was localized between markers NAU5303 and NAU3918 (3,545,485–8,213,231 bp)*.* Cluster, *C7-m-cluster-2* from the 40–58 cM interval was located between markers BNL1597 and NAU2186 (9,280,354–15,534,308 bp). Cluster, *C7-m-cluster-3* from 60 to 72 cM interval was situated between markers NAU1085 and CIR238 (16,350,941–2,178,086 bp). The cluster on Cons.c14, *C14-m-cluster-1,* from 0 to 15 cM interval was localized between markers SWU14174 and SWU14188 (17,025,534–21,454,750 bp)*.* Cluster*, C14-m-cluster-2* from 20 to 36 cM interval was localized between markers BNL3099 and COT099 (49,640,545–50,515,032 bp)*. C14-m-cluster-3* from 38 to 54 cM interval was between markers NAU3393 and PGML0989 (11,844,310–17,029,745 bp) and *C14-m-cluster-4* from the 58–78 cM interval was between markers DPL0354 and BNL3033 (62,556,024–70,746,352 bp).

## Discussion

### Genetic map

The identification of stable QTLs for superior agronomically significant traits and the construction of a high-resolution map are essential for MAS. Several intraspecific genetic maps have been reported; however, these contain some gaps that limit its applicability in generating a high-density genetic map. Major obstacles in the construction of a high-resolution map in intraspecific crosses include a low rate of polymorphism within *G. hirsutum* and the presence of fixed homozygous genetic blocks [23]. Therefore, there is a need to identify additional markers that covers these gaps in the genetic map. In the present study, an updated genetic map based on our previous report showing 190 markers [18] is described. We have added 586 markers including 386 (41 % of the total number of markers) novel SWU primers. Among these 793 markers, 524 were mapped to the D_t_ sub-genome and 269 were mapped to the A_t_ sub-genome. In our previous report, chromosomes c4, c7, c13, c14, c18, and c25 were identified as important and rich in QTLs for fiber quality traits [18]. Most of the new markers that we have successfully added to the map have been localized to these chromosomes, thereby enabling us to dissect these QTLs into clusters at a higher resolution, as well as identify some important stable QTLs for specific superior features. In the current map, 20 chromosomes harbored more than one linkage group, which indicates a relatively low rate of polymorphism in intra specific crosses which was observed at a rate of 2.9 % in the present study. The observed relatively low rate of polymorphism suggests that the genetic distance between the two parents was very narrow, thereby indicating the need for a saturated intra-specific map. Therefore, our next goal is to develop new SSR and SNP primers that would facilitate in the construction of a saturated genetic map.

### Segregation distortion

Among the 793 mapped primers, 361 showed distortion from the normal Mendelian ratio, which is 1:1 in the case of RILs. This severe distortion was also reported by Sun et al. [18] and commonly occurs in RIL populations that were developed from an introgressed line parent. This high ratio of segregation distortion in our population may be attributed to parent 0–153, which is an introgressed line. Tang et al. [33] also reported similar results (41.8 %) in their RIL population with introgressed parental line, 7235. Segregation distortion could be influenced by various factors including genetic factors such as genetic drift [45] and the environment. However, it does not significantly impact the estimation of QTL position and effect [46]. The broad sense heritability estimates of fiber quality traits were high for FL, FM, FS and FU, indicating that the QTLs identified in this population are more reliable and useful in MAS for cotton breeding.

### Distribution of QTLs among A_t_ and D_t_ sub-genomes

The distribution of QTLs was not uniform in the A_t_ and D_t_ sub-genomes. Among the 165 QTLs identified, 58 QTLs (35 % of the total) were identified in the A_t_ sub-genome, whereas 107(65 % of the total) were identified in the D_t_ sub-genome. Previous comparative meta-analyses conducted by Rong et al. [32], Lacape et al. [43] and Said et al. [36] have indicated that in cotton a higher number of QTLs for fiber traits resided within D_t_ sub-genome chromosomes, and gene expression among homologous pairs were not uniform [44, 47]. Yu et al. [48] also observed 35 % more QTLs in the D_t_ sub-genome in an inter specific backcross inbred line population. In the present study a higher number of loci were mapped to the D_t_ sub-genome. This observation might be due to the presence of more SSR markers that were developed from the D genome sequence [26], although this phenomenon has also been previously described by Yu et al. [49] in their BC_1_ population. However we also observed that some A_t_ sub-genome chromosomes also have more loci than its homologous counterparts in D_t_ sub-genome chromosomes. This unequal distribution of loci indicates the presence of active regions with more recombination frequencies in the upland cotton genome [4]. Similarly, QTLs on both pairs were also not homogeneous. Most importantly, homology was observed between homologous pair c6-c25 and c7-c16, which harbored QTL clusters and were in agreement with the findings of previous reports [23, 43].

Comparison of the tetraploid cotton genome with its ancestors shows that only the A genome (*G. arboreum*) produces spinnable fibers, whereas the D genome (*G. raimondii*) lacks this characteristic. After polyploidization, transposable elements tend to be more active, especially in the D_t_ sub-genome, compared to that in the A_t_ sub-genome. Furthermore, the D_t_ sub-genome also has a higher mutation rate than the A_t_ sub-genome [28]. These findings might also contribute to our observation that the D_t_ sub-genome harbored more QTLs than the A_t_ sub-genome. However, the additional of novel markers for the A_t_ Sub-genome may improve the assessment of the contribution of each sub-genome in fiber quality traits.

### Consistency with previously reported fiber QTLs

It is very difficult to compare different QTLs that have been reported in various populations, although this is necessary to fully understand the behavior of complex traits, particularly in a changing environment. In present study, 325 markers were designated as novel SSRs (Additional file 8). However, some regions did not have common markers at QTLs and thus we were unable to compare these with the findings of previous reports. However some stable QTLs with common markers have been identified and were used in our meta-analyses. We identified 38 cluster regions. When a meta-cluster contained stable QTLs from our RIL population and QTLs were identified by recent meta-analyses report [35], this was considered as the same cluster. We also confirmed the previous meta-analyses report [35], which in turn allowed us to declare a true stable QTL in this consensus genomic region. For example Lacape et al. [12], Shen et al. [5, 6] and Sun et al. [18] reported QTLs for fiber strength and length that were linked to primers BNL3806, TMK19, and BNL1440 on c25. We have identified two clusters that were tightly linked to these primers. Four QTLs for fiber quality traits FE, FL, FM and FS were closely linked to primer BNL3806 and TMK19. Four QTLs for the fiber quality traits, FE, FL, FS, and FU were tightly linked to BNL1440. These QTLs were in two meta-cluster regions *C25-cluster-1*:*0*–*20 cM* and *C25-cluster-2-25-45 cM*. Our results confirm the findings of Said et al. [36] as well as declare that these QTLs are indeed stable. We also verified its physical position in the genome sequence of *G. hirsutum*. QTL analysis on the basis of the physical map also confirmed that these loci were closely linked to these fiber quality traits. However, additional studies confirming the presence of putative genes in this region are warranted. Meta-clusters that harbor QTLs from our RIL population and the latest QTL studies except for those identified by Said et al. [36] were regarded as new meta-clusters in the present study. Of the 38 meta-clusters, 31 clusters with 314 QTLs were considered similar to that of a previous report [36]. In Addition, we identified seven novel cluster regions with 55 QTLs for fiber quality traits in the present study. The cluster on Cons.c4, C*4-m-cluster-1,* which contained 14 QTLs including five fiber quality traits FE, FS, FL, FU, and FM was considered as novel. Three stable QTLs identified in our RIL population *qFE-C4-3, qFM-C4-2,* and *qFL-C4-2* and one stable QTL identified by Tang et al. [33], *qFS04.1* were also detected in this cluster region. The cluster on c7, *C7-m-cluster-3* which contained six QTLs for three fiber traits FL, FS, and FU was considered as a novel cluster. One stable QTL, *qFS-C7-2*, which was identified in our RIL population and one QTL, *qFU07.1* identified by Tang et al. [33], were also confined in this cluster region. On Cons.c14, *C14-m-cluster-2* and *C14-m-cluster-3* were respectively identified as novel clusters. The *C14-m-cluster-2,* contained 16 QTLs including six stable QTLs for five fiber quality traits, were identified in our RIL population. *C14-m-cluster-3* contained three stable QTLs that were identified in our RIL population and one stable QTL *qFS14.1,* that was earlier identified by Tang et al. [33]. On c15 and c20, *C15-m-cluster-4 and C20-m-cluster-3* were considered as novel clusters, respectively (Additional file 5). On c25, *C25-m-cluster-4* which contained six QTLs for fiber quality trait was considered as a novel cluster. Fine mapping of c25 was also performed and discussed separately [50].

## Conclusion

QTLs detected in different environments are stable QTLs [51], that may be utilized in MAS and RIL population are useful in the detection of stable QTLs in multiple environments [52]. We have identified 165 QTLs, of which 30 QTL clusters were identified in an intraspecific RIL population in 11 environments. Meta analyses results have revealed that 90 fiber QTLs in the RIL population were in agreement with the findings of previous reports. We have identified seven novel cluster regions that contained 55 fiber QTLs, including 33 QTLs from the RIL population. QTL clusters on c4, c7, c14 and c25 were identified as stable across multiple environments and populations. Therefore, these clusters were considered important for cotton breeders and can be utilized in MAS to improve fiber quality.

## Methods

### Mapping population

A segregation population consisting of 196 F_6:8_ RIL individuals were derived from a cross between two upland cotton strains, 0–153 and sGK9708. Strain sGK9708 is insect resistant with moderate fiber quality and high yield potential, whereas strain 0–153 has excellent fiber quality with low yield. The cross was made in 2001 and recombinant inbred lines were developed as detailed by Sun et al. [18]. From 2007 to 2013 multi-environmental evaluations were conducted in six different locations throughout China with two replications in each environment (Table 1). Sun et al. [18] reported four environments from the year 2007 to 2008. We added seven more environments with three additional locations to the total phenotypic data set (Table 1). These evaluation procedures were also earlier described by Zhang et al. [50].

### Phenotyping

Fiber samples were collected from each line to investigate fiber quality traits. 30 normally opened bolls were collected from each plot. Fiber quality traits were measured using an HVI-100 instrument (user technologies, Switzerland) at the Cotton Fiber Quality Inspection and Testing Center of Ministry of Agriculture, Anyang, China. The fiber quality traits included FE, FL, FM, FS and FU. These observed phenotypic data were analyzed by using the software SPSS20.0 (SPSS, Chicago, IL, USA). For ANOVA, we used the SAS statistical software (version 8.1; SAS institute, Cary NC). To calculate broad sense heritability the following equation was used

$$ {{\mathrm{H}}^2}_{\mathrm{B}}\left(\mathrm{Broadsenseheritability}\right)={\upsigma}^2\mathrm{G}/\left(\upsigma \mathrm{G}+{\upsigma}^2\mathrm{G}\ast \mathrm{E}/{\mathrm{n}}_{\mathrm{e}}+{\upsigma}^2\mathrm{E}/{\mathrm{n}}_{\mathrm{e}}{\mathrm{n}}_{\mathrm{r}}\right) $$

Where σ^2^G is genotypic variance, σ^2^G*E is genotype * environment variance, and σ^2^E is variance of error.

### Genotyping

#### DNA extraction

Young leaves were collected from each line and stored at −80 °C. Genomic DNA from the parents and 196 RILs was extracted using a modified CTAB method as described by Paterson *et al.* [53]. PCR amplification was performed in a total reaction volume of 10 μL containing 6.15 μL ddH_2_O, 1 μL 10× buffer (with 1.5 mL Mg^+^), 0.5 μL dNTPs (10 mM), 0.5 μL each primer, 0.15 μL of *Taq* polymerase (500U) and 1.2 μL of genomic DNA (30 ng/μL). PCR amplification conditions comprised of an initial denaturation at 95 °C for 3 min, followed by 30 cycles of denaturation at 94 °C for 1 min, annealing at 57 °C for 30s and an extension at 72 °C for 60s followed by a final elongation at 72 °C for 5 min, and then held at 4 °C until analysis. PCR products were electrophoresed on an 8 % non-denatured polyacrylamide gel and silver staining was used for visualization of bands.

#### SSR analyses

A total of 28,891 primers pairs, including 12,560 SWU primers (D genome sequence-based), were used to detect polymorphisms between the two parents. Approximately 851 polymorphic primers were selected and used in genotyping 196 recombinant inbred lines. All loci were named according to their respective primer names. In the case of multiple loci generated by single primer pair that showed a different segregation pattern from that of the main band, a suffix of a/b/c was used after the primer name to differentiate loci according to increasing molecular size. The details of the primers used in the present study are listed in Additional file 7. The SWU primers were synthesized by Beijing Genomics Institute (Beijing, China), whereas all other primers were synthesized by Invitrogen, Co. Ltd. (Shanghai, China) and Bio Asia, China (Beijing, China).

#### Construction of the genetic map and QTL analyses

A linkage map was constructed using JoinMap 4.0 [54] with a logarithm of odds (LOD) threshold of >7 and a maximal distance of 50 cM. Recombination frequencies were converted to map distance using the Kosambi map function [55]. For some groups that have mixed markers belonging to different chromosomes, a higher LOD score of >9 was used to separate these into small groups. Linkage groups were assigned to its respective chromosome based on previous reports [18, 19, 20, 33, 56,57] and marker mapping information from the CottonGen database (http://www.cottongen.org/). Small groups that were mapped to the same chromosome were recalculated to combine these into one group. A minimum LOD score of 6 was used to combine these groups. In the case of c20 and c23, an LOD score of 5 was used to combine small linkage groups into one. The *G. hirsutum* fasta sequence was downloaded from http://www.cottongen.org/ and used to check co-linearity of loci between the linkage map and the *G. hirsutum* physical map.

#### QTL analyses and meta-analysis

Windows QTL Cartographer 2.5 [57] was used for QTL mapping. The composite interval mapping method [42] was used at a walking speed of 1 cM and using a 1000-permutation test. QTLs for the same trait across different environments were declared the same when its confidence interval overlapped. A QTL identified in at least three environments was declared as stable. We used a physical map in which loci were arranged according to their position on the *G. hirsutum* genome, and QTL analysis was performed using the composite interval mapping method as earlier described.

Meta-analysis was performed with Biomercator 4.2 [34] as described elsewhere [36]. A previous meta-QTL analyses established a QTL data-base [35] consisting of 2,274 QTLs, which included 437 highly consistent QTLs for fiber quality traits from 58 QTL reports on upland cotton [35]. We downloaded its QTL information, including names and CI from www.cottonqtldb.org. We used the high-density consensus map [58] as a reference to project our QTLs and performed chromosome-wise meta-analyses. A total of 850 fiber QTLs from six QTLs reports including 165 fiber QTLs from our RIL population, 50 fiber QTLs from the F_2_,F_2:3_, and RIL populations of same parents [18], and 635 fiber QTLs from previous reports literatures [33, 35, 37, 38] were thereby generated.

For meta-analyses, two separate input files were prepared, a map file and a QTL file. The map file contained distances between markers on each chromosome, and the QTL file contained 12 columns, where each row represented a single QTL in a given environment, i.e., QTL name, trait name, trait ontology, experiment place, year, chromosome name, linkage group name, LOD score, observed PV value (R2), most likely position of the QTL, CI start position and CI end position. First both files were loaded into the software and checked for map connectivity. Then QTLs were projected on a consensus map and meta-analyses were performed for each trait. Four models were thus generated, each with an Akaike information criterion (AIC) value. The model with lowest AIC value was selected and used for the identification of mQTL position, whereas QTL clusters were determined manually. The QTLs within the region of 20 cM on the consensus map were considered as part of same cluster as earlier defined by Said et al. [36].

## Additional files

Additional file 1: Genetic linkage map of an intraspecific RIL population. (ZIP 7480 kb) Additional file 2: Fiber QTLs identified in an intraspecific RIL population and their position. (XLSX 39 kb) Additional file 3: Position and markers details of identified QTLs for fiber quality traits in an intraspecific RIL. (XLSX 37 kb) Additional file 4: Details of QTL clusters on the 0–153 genetic map of a RIL. (XLSX 29 kb) Additional file 5: Summary of meta-clusters on a consensus map. (XLSX 10 kb) Additional file 6: Identified QTLs for fiber traits in a RIL and comparison with the findings of previous reports. (XLSX 46 kb) Additional file 7: Meta-analysis results of the remaining chromosomes. (DOCX 672 kb) Additional file 8: Details of the primers used in the study. (XLSX 10 kb)

## Abbreviations

AIC: Akaike information criterion
c: chromosome
CI: confidence interval
FE: fiber elongation
FL: fiber length
FM: fiber micronaire
FS: fiber Strength
FU: fiber uniformity
H^2^_B_: broad sense heritability
LOD: logarithm of odds
MAS: marker-assisted selection
PV: phenotypic variance
QTL: quantitative trait loci
SDRs: segregation distortion regions
SSR: simple sequence repeats
