# Supplementary figures and images for: Identification of stable quantitative trait loci (QTLs) for fiber quality traits across multiple environments in Gossypium hirsutum recombinant inbred line population
Source: BMC Genomics. 2016 Mar 8;17:197. doi: 10.1186/s12864-016-2560-2 (PMC4782318; doi:10.1186/s12864-016-2560-2)

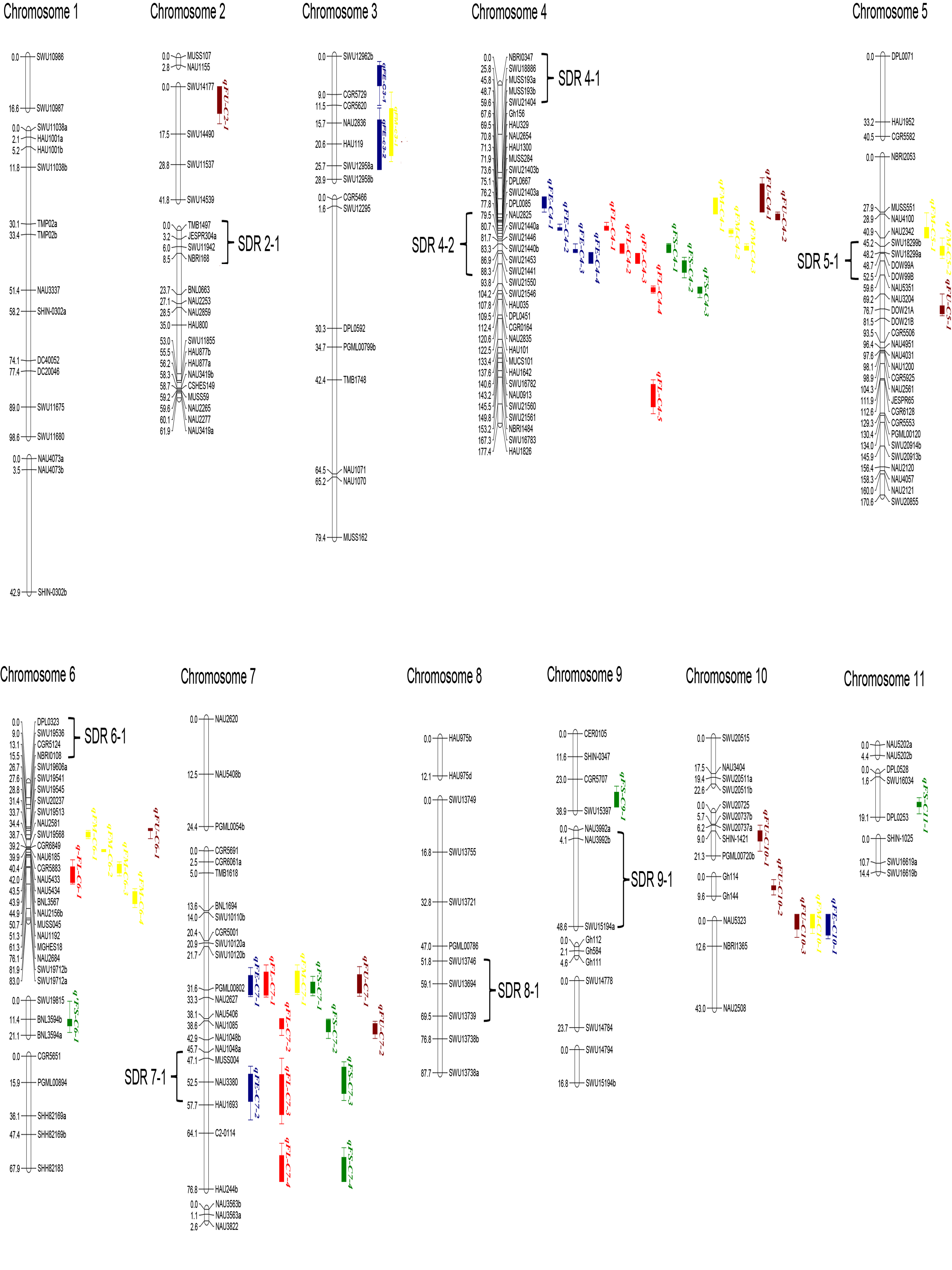

Supplement: Additional file 1: — Genetic linkage map of an intraspecific RIL population. (ZIP 7480 kb) [file 12864_2016_2560_MOESM1_ESM.zip › Additional file 1, Figure S1a 1.tif]

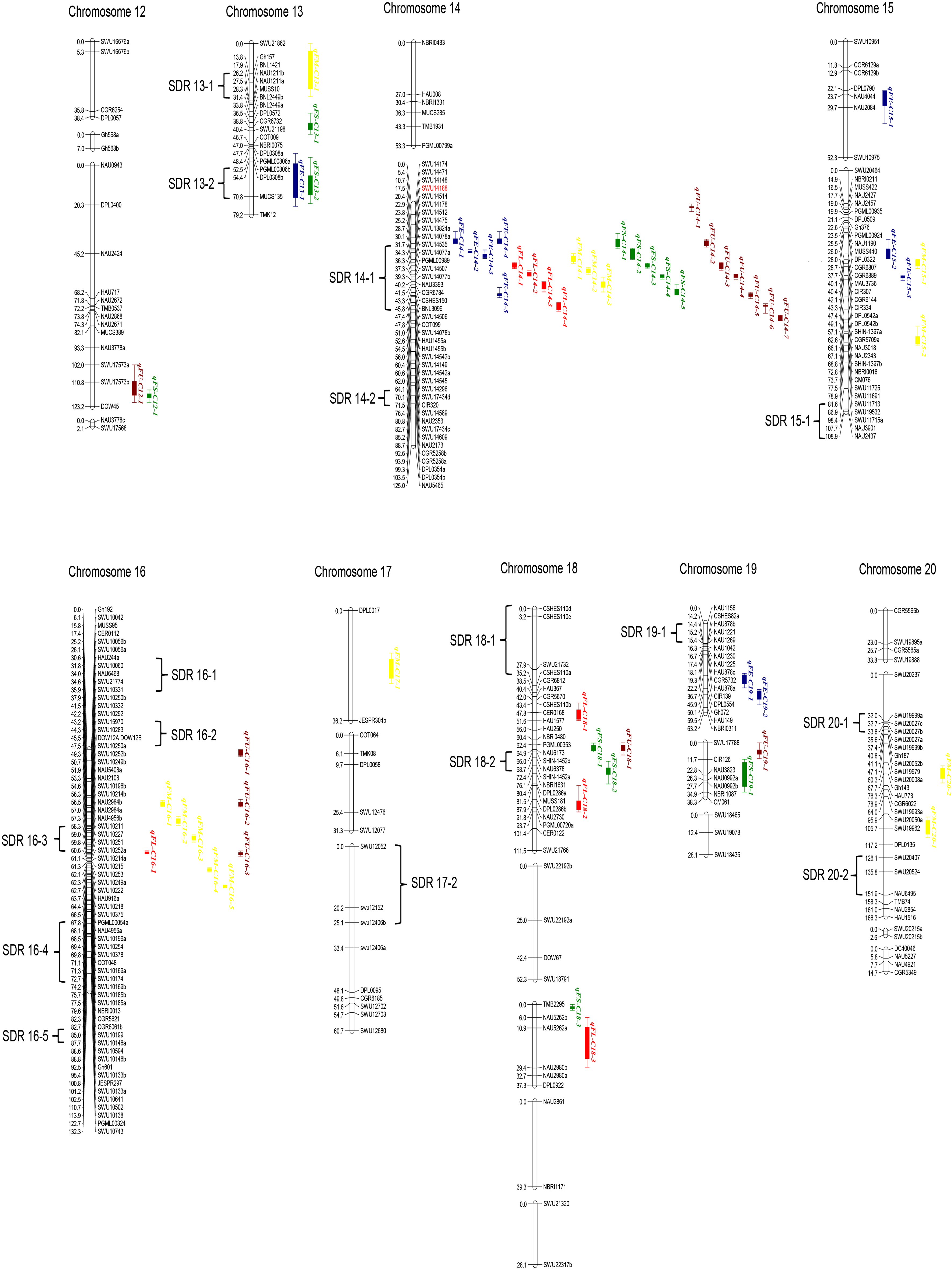

Supplement: Additional file 1: — Genetic linkage map of an intraspecific RIL population. (ZIP 7480 kb) [file 12864_2016_2560_MOESM1_ESM.zip › Additional file 1, Figure S1a 2.tif]

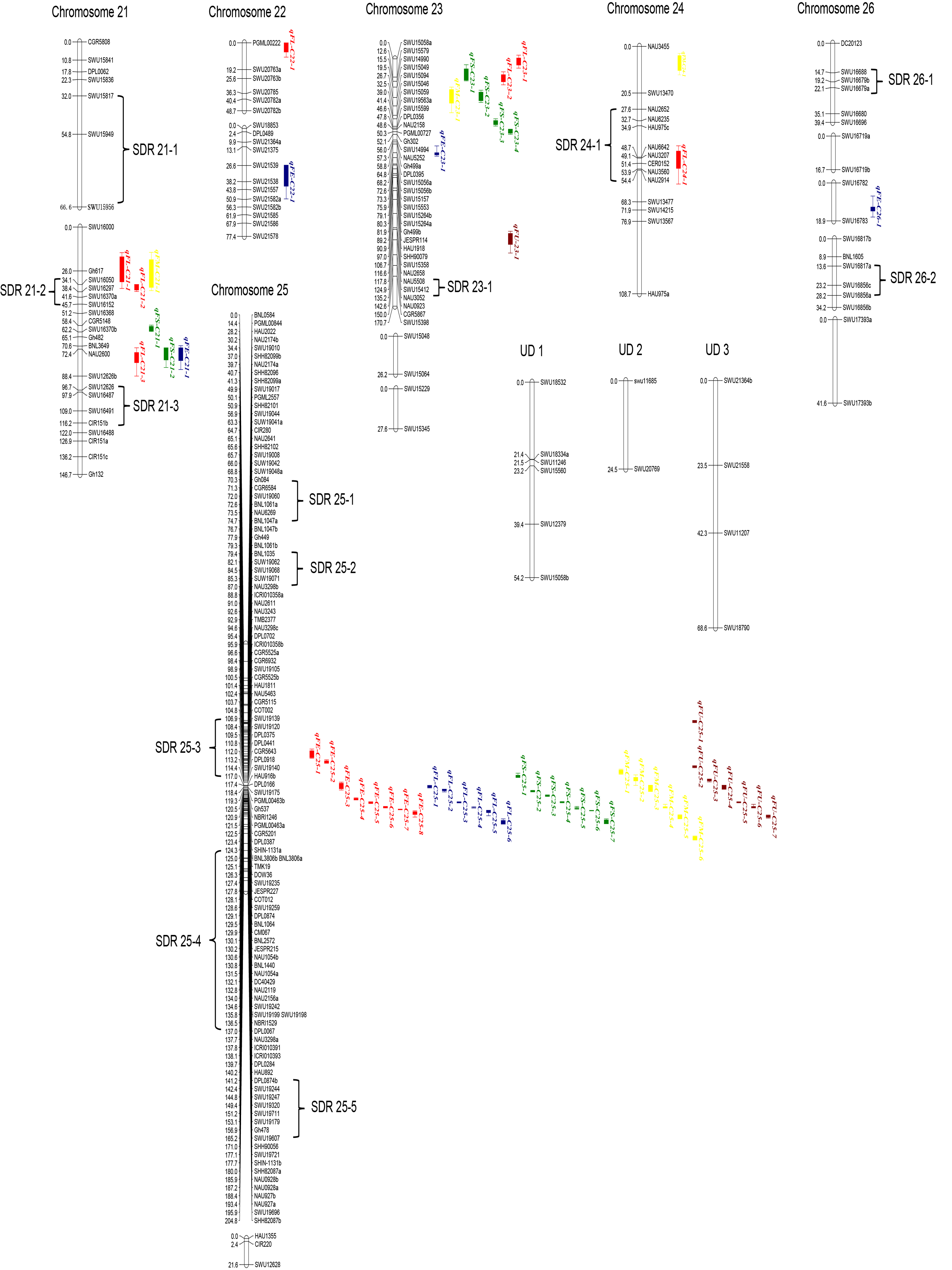

Supplement: Additional file 1: — Genetic linkage map of an intraspecific RIL population. (ZIP 7480 kb) [file 12864_2016_2560_MOESM1_ESM.zip › Additional file 1, Figure S1a 3.tif]
