# Supplementary material for: Identification of stable quantitative trait loci (QTLs) for fiber quality traits across multiple environments in Gossypium hirsutum recombinant inbred line population
Source: BMC Genomics. 2016 Mar 8;17:197. doi: 10.1186/s12864-016-2560-2 (PMC4782318; doi:10.1186/s12864-016-2560-2)
Supplement: Additional file 7: — Meta-analysis results of the remaining chromosomes. (DOCX 672 kb) [file 12864_2016_2560_MOESM7_ESM.docx]

**Additional file 7:** Meta-analysis results of the remaining chromosomes

Chromosome 5 contained four clusters.*C5-cluster-1 0-18cM* contained 8 QTLs, *C5-cluster-2 :20-38cM* contained 4QTLs, *C5-cluster-3:40-58cM* contained 2 QTLs and *C5-cluster-4* :60-78cM contained 3 clusters on consC5.Two hotspotes were found one for FE (0-18cM) and other for FM (22-38cM)


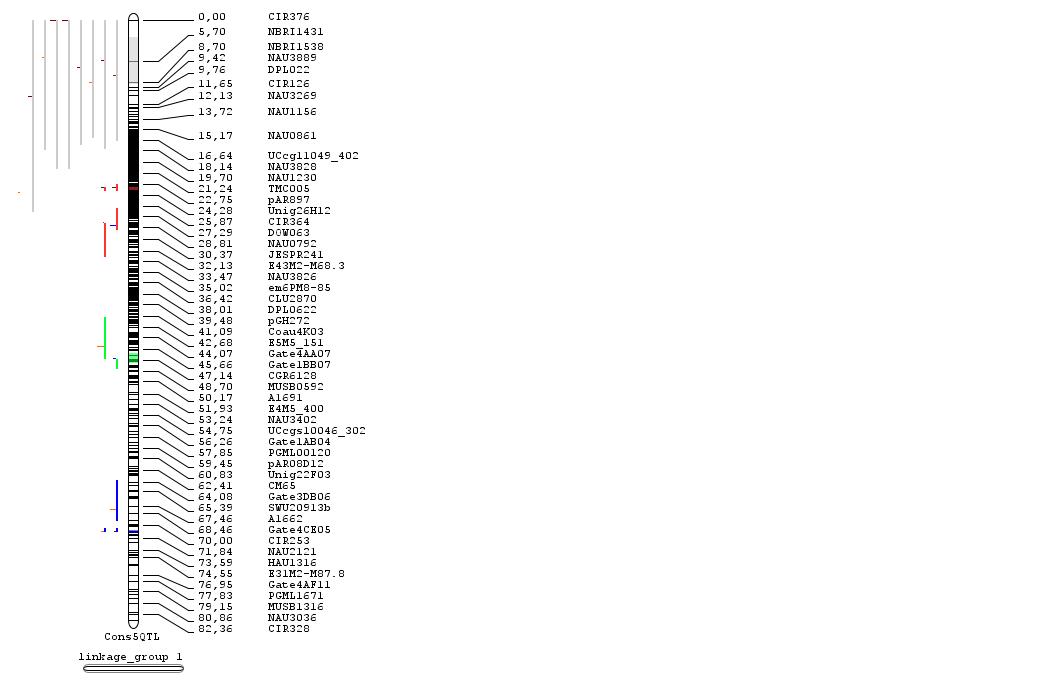


Chromosome 12 had 4 QTL clusters. *C12-cluster-1:0-20cM contained* 11QTLs, *C12-cluster-2:22-40cM* contained 19 QTLs. *C12-cluster-3:42-60cM* contained 13 QTLs and *C12-cluster-4:62-82cM* contained 4 QTLs for fiber traits.

d
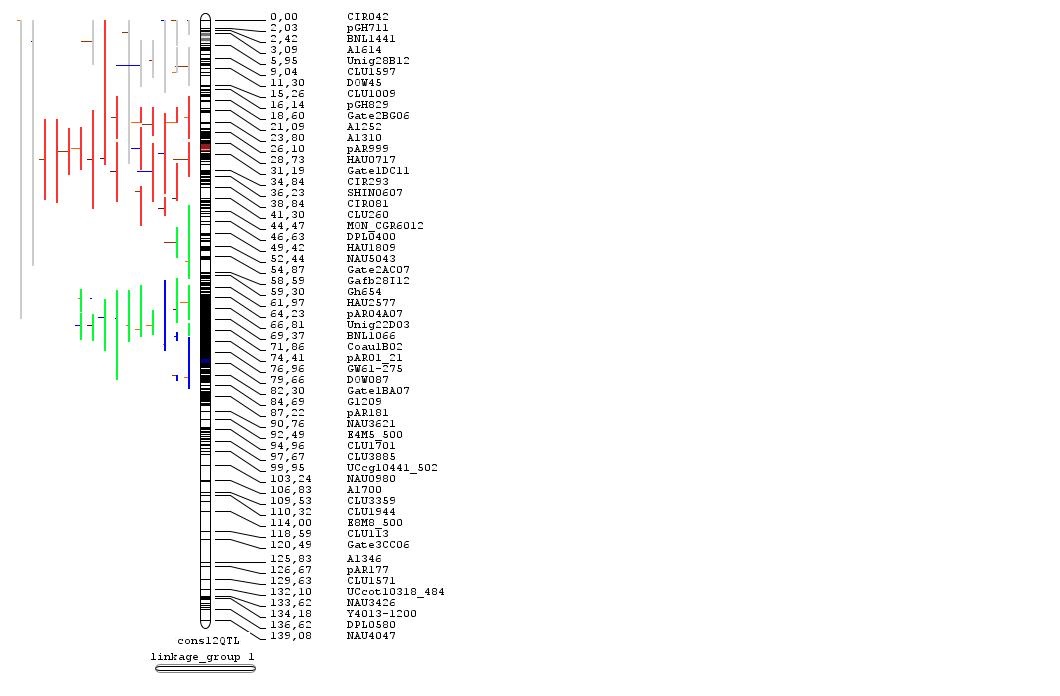


Chromosome 13 had 3 clusters. *C13-cluster-1:40-58cM* contained 5 QTLs*, C13-cluster-2 :60-76cM* contained 13 QTLs and *C13-cluster -380-100cM* contained 4 QTLs for fiber traits*.
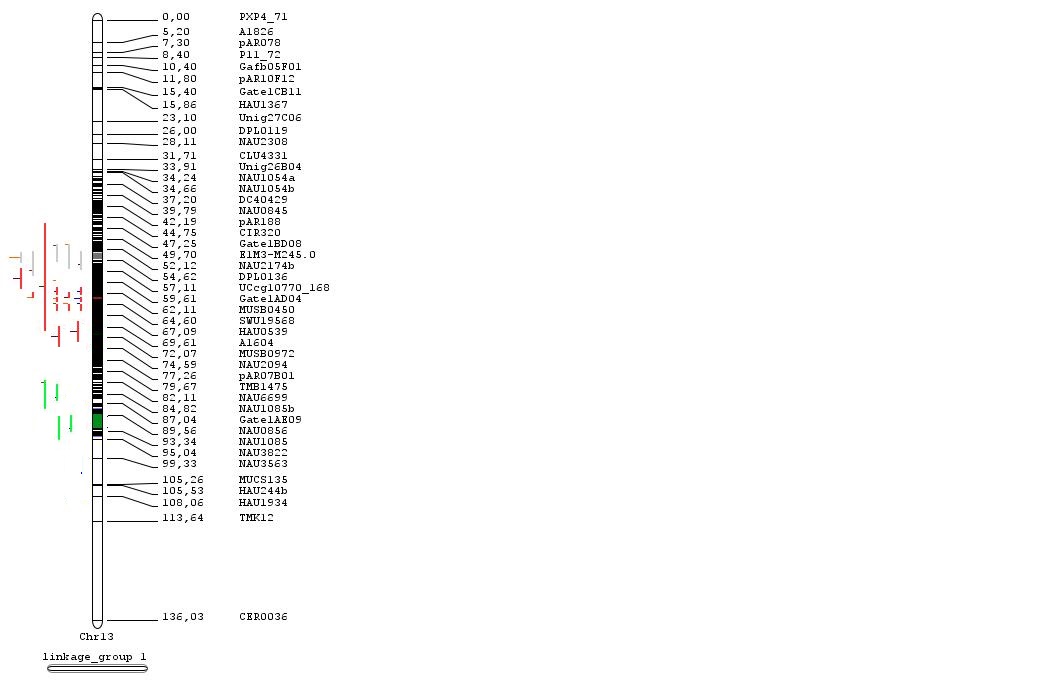
*

Chrmosome 15 had 4 QTL clusters*. C15-cluster-1:23-44cM* contained 7 QTls, *C15-cluster-2:46-64cM* 4QTLs, *C15-cluster-3:67-*82cM contained 7 QTLs and C15*-cluster-4:155-170cM* contained 3 QTLs for fiber quality traits.


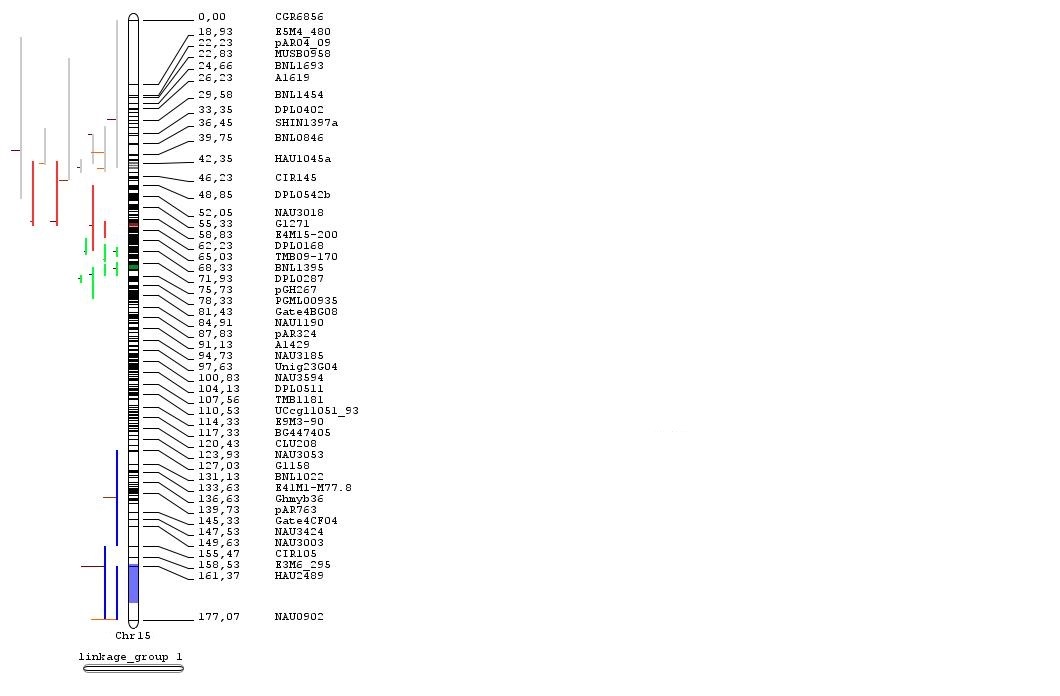
Chromosome 16 had 3 clusters. *C16-cluster-1:9-29cM* contained 15 QTLs, *C16-cluster-2:50-70cM contained* 11 QTLs and *C16-cluster-3:75-95cM* contained 13 QTLs.


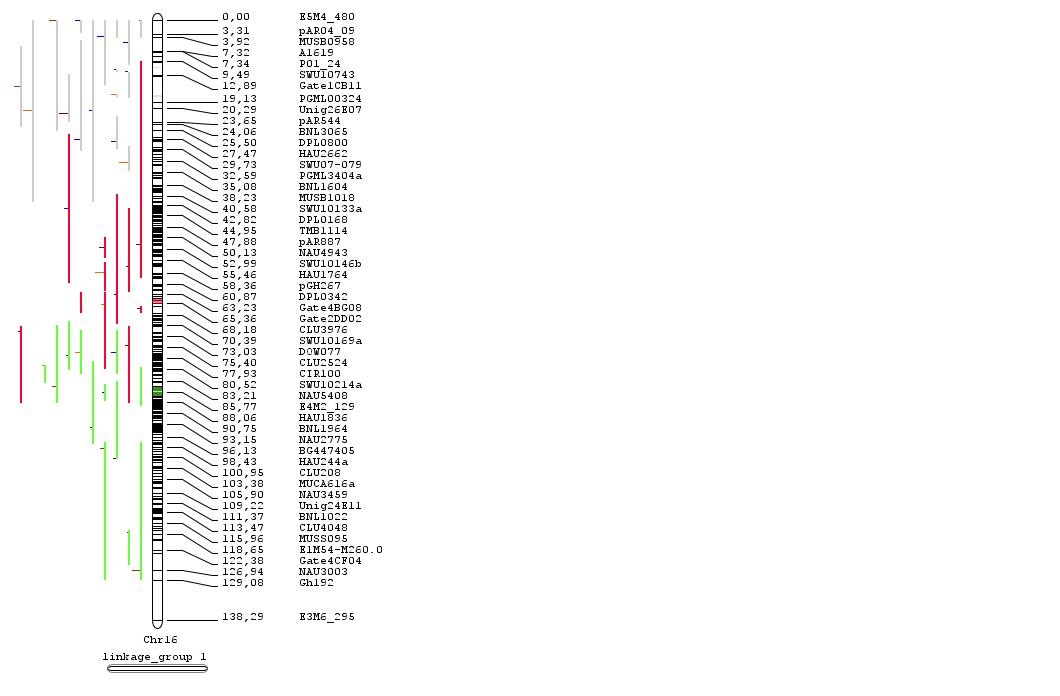


Chromosome 20 had 3 clusters. *C20-cluster-1:22-53cM* contained 3 QTLs, *C20-cluster-2:73-93cM contained* 8 QTLs and *C20-cluster-3:125-135cM* contained 2QTLs.


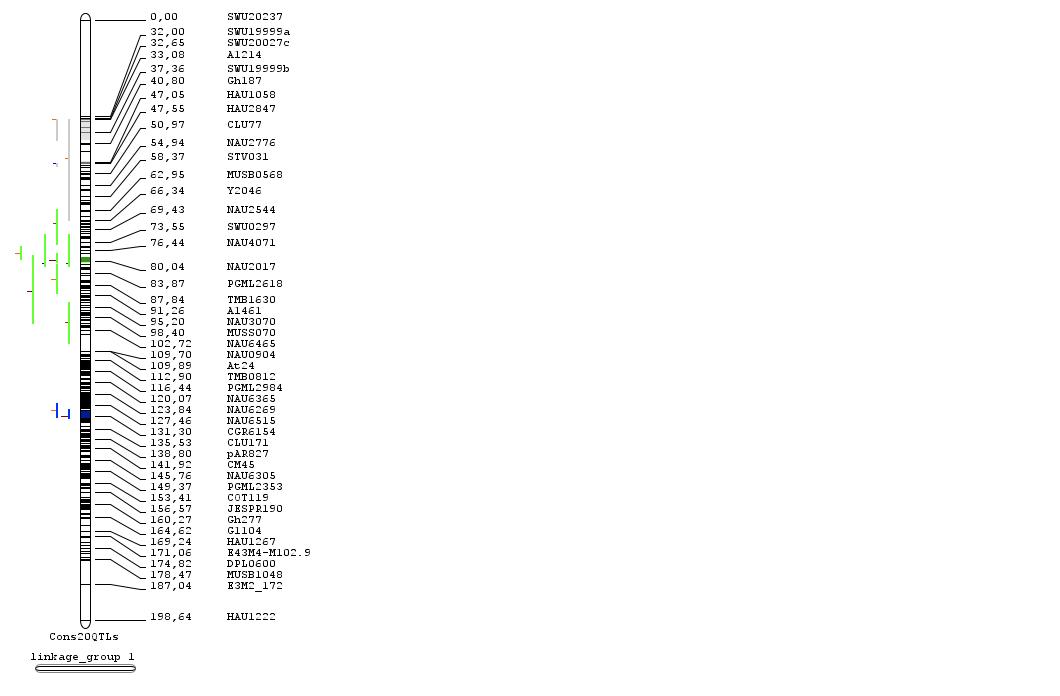


Chromosome 23 had 4 clusters. *C23-cluster-1:20-40cM* contained 14 QTLs, *C23-cluster-2:40-60cM contained* 13 QTLs and *C23-cluster-3:80-100cM* contained 9QTLs. *C23-cluster-4:140-160cM* contained 8 QTLs. All four cluster regions confirmed previous report.


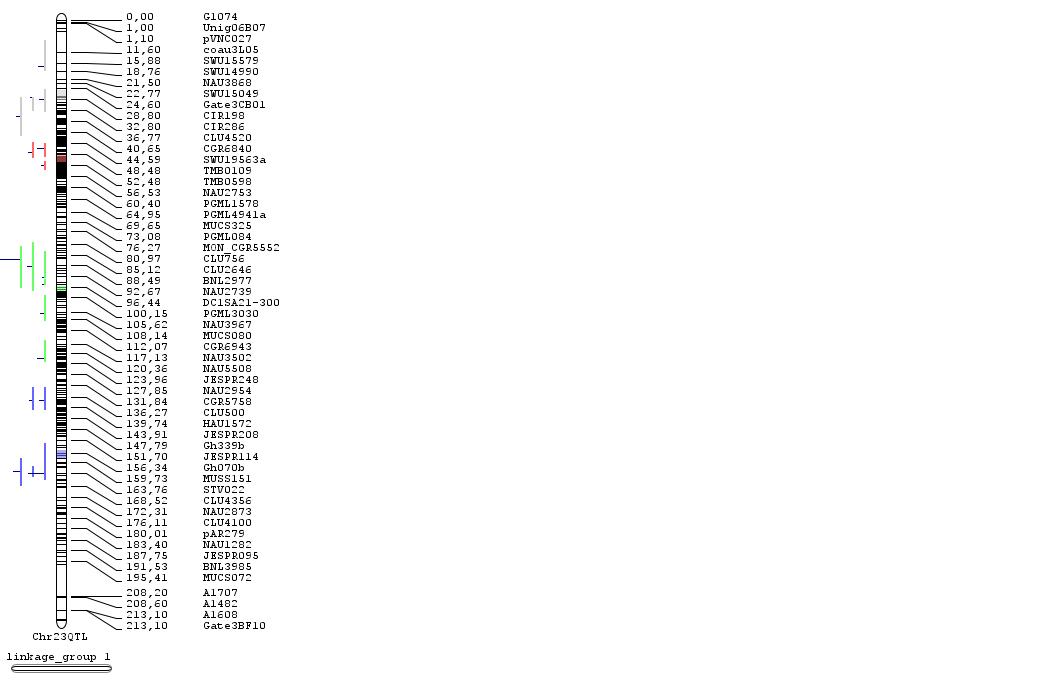


Chromosome 25 had 4 clusters. *C25-cluster-1:0-20cM* contained 18QTLs, *C25-cluster-2:25-45cM contained* 15 QTLs and *C25-cluster-3:50-70cM* contained 21QTLs. *C25-cluster-4:71-90cM* contained 6 QTLs. First 3 cluster regions confirmed previous report while cluster four with 6 QTLs is novel cluster


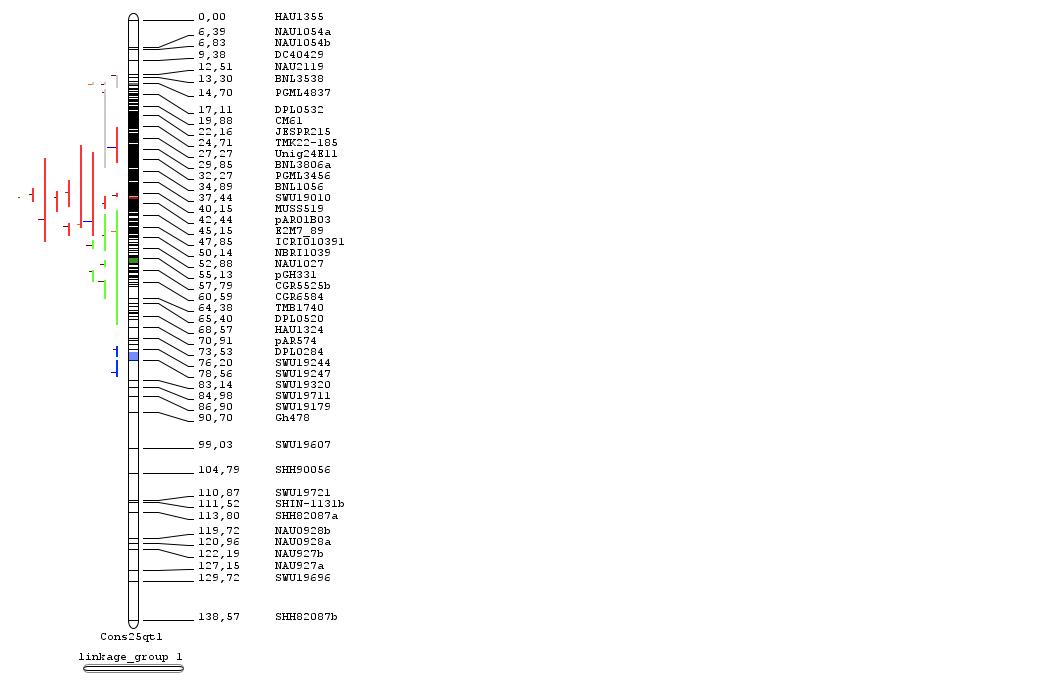

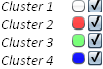


Clusters were represented with colors. Cluster 1 is represented by grey, Cluster 2 with Red, cluster 3 with green and cluster 4 with blue
